# Invisible and Visible Processing of Facial Attractiveness in Implicit Tasks: Evidence From Event‐Related Potentials (ERPs)

**DOI:** 10.1002/pchj.70014

**Published:** 2025-04-23

**Authors:** Junchen Shang, Kaiyin Zhong, Xuejiao Hou, Liansheng Yao, Rui Shi, Zuo‐jun Wang

**Affiliations:** ^1^ Department of Medical Humanities, School of Humanities Southeast University Nanjing China; ^2^ College of Psychology Liaoning Normal University Dalian China; ^3^ College of Education Suihua University Suihua China; ^4^ State Key Laboratory of Brain and Cognitive Science Institute of Psychology, Chinese Academy of Sciences Beijing China; ^5^ Department of Psychology University of Chinese Academy of Sciences Beijing China; ^6^ School of Economics and Management Yanshan University Qinhuangdao China; ^7^ School of Educational Science Anhui Normal University Wuhu China

**Keywords:** awareness, continuous flash suppression (CFS), event‐related potentials (ERPs), facial attractiveness, implicit processing

## Abstract

Facial attractiveness can be automatically perceived in implicit tasks when the faces are visible. Nonetheless, to date, it is poorly understood to what extent facial attractiveness can be processed when faces are invisible. It is also worth exploring the differences between visible and invisible processing of facial attractiveness. To address these issues, the event‐related potentials (ERPs) were recorded when participants were presented with attractive and unattractive faces under invisible condition (continuous flash suppression paradigm; CFS) and visible condition (gender judgment task). The results indicated that attractive faces elicited a larger P1 amplitude (110–150 ms) compared to unattractive faces, regardless of whether the faces were visible. Attractive faces also elicited a larger N170 amplitude (150–190 ms) compared to unattractive faces under the visible condition. Furthermore, visible faces elicited larger P1 and N250/early posterior negativity (EPN) amplitudes as compared to invisible faces. But only under the attractive condition, the visible faces elicited a larger N170 than the invisible faces. The present study suggested that facial attractiveness can be automatically perceived in the early stage regardless of visibility, although attractiveness processing was somewhat reduced in the absence of visual awareness.

## 1 Introduction

Perception of faces is an important visual skill humans possess since facial information plays a pivotal role in human life. When we encounter a face, we process at least two primary types of information (Jiang et al. 2009). Firstly, a face is recognized as such, including both a general facial category and a unique facial identity. Secondly, other information may be interpreted in relation to its potential relevance to evolution. Facial attractiveness plays an important role in mate choice (e.g., Rhodes 2006) as it is associated with genetic quality and longer life span (Hahn and Perrett 2014). There are prosocial biases in favor of attractive adults in everyday life. For example, attractive adults are more likely to win elections (Stockemer and Praino 2017) and be hired in the labor market (Dipboye et al. 1977; Hamermesh and Biddle 1994). More interestingly, the processing of facial attractiveness is automatic (Hung et al. 2016).

On the one hand, attractive faces evoked significant event‐related potential (ERP) differences compared to unattractive faces when participants were asked to perform implicit tasks irrelevant to explicit evaluation of attractiveness, for example, to judge face gender (Schacht et al. 2008; Wiese et al. 2014), to make economic decisions relevant to the faces (Chen et al. 2012; Jin et al. 2017; Ma et al. 2015, 2017; Pei and Meng 2018), to passively view faces (Halit et al. 2000; Johnston and Oliver‐Rodriguez 1997; Muñoz and Martín‐Loeches 2015; Oliver‐Rodríguez et al. 1999), to disengage attention from the faces (Van Hooff et al. 2011), or to recall the faces (Marzi and Viggiano 2010; Zhang et al. 2011, 2016).

On the other hand, facial attractiveness can even be extracted when the faces are rendered invisible using the masking paradigm or the continuous flash suppression (CFS) paradigm, though controversies still exist. Research using the masking paradigm involves presenting a random noise or an unrelated visual image, either before or after a briefly presented stimulus. For example, Olson and Marshuetz (2005) found that people can perceive facial attractiveness even if the faces were only presented for 13 ms and masked, indicating that the processing of facial attractiveness does not require consciousness. Hou et al. (2023) presented the faces for only 10 ms and similarly found that facial attractiveness was extracted. However, Tsikandilakis et al. (2019) presented the faces for 33.33 ms, where participants were asked to judge whether the image they saw was a face. Results showed that participants could correctly identify facial attractiveness when they could see it, but they were unable to judge attractiveness when they could not see it. They argue that conscious awareness is a prerequisite to evaluate facial attractiveness. The CFS paradigm is a modified binocular rivalry technique that can render a stimulus invisible for several seconds, placing it at the unconscious level (Tsuchiya and Koch 2005). It works by presenting a series of high‐contrast noise images to the dominant eye, suppressing the entry into consciousness of a low‐contrast stimulus presented to the non‐dominant eye. For example, researchers have found that in the b‐CFS paradigm (a variant of the CFS), attractive faces gain visual dominance and enter consciousness faster than unattractive faces (Hung et al. 2016; Nakamura and Kawabata 2018), suggesting that attractiveness can be processed in the absence of visual awareness. However, other scholars argue that reaction times in b‐CFS merely represent the transition speed from unconscious perception to conscious perception (Del Río et al. 2018). Thus, it remains unclear whether facial attractiveness can be processed without conscious awareness. Additionally, behavioral research limited understanding of the neural mechanism of unconscious attractiveness processing, such as the temporal dynamics of attractiveness information extraction when faces were rendered invisible.

To sum up, although previous studies have found that the implicit processing of facial attractiveness is automatic when the faces are visible, there is still controversy over whether the automatic processing of facial attractiveness depends on conscious awareness of the faces. The current study investigated whether conscious perception is a necessary condition for the implicit and automatic processing of facial attractiveness. If facial attractiveness can be processed to the same extent when they are rendered completely invisible as when the faces are visible, it may help to explain why human society is susceptible to the attractiveness of faces.

This research employed electroencephalography (EEG) techniques in a CFS task and a gender judgment task to examine the disparities in the implicit processing of facial attractiveness between invisible and visible conditions. On the one hand, we examined whether attractive faces evoked significant ERP differences compared to unattractive faces with and without influence from a conscious representation. Previous studies revealed that when faces are visible, facial attractiveness is associated with various ERP components depending on different tasks. Zhang and Deng (2012) asked participants to detect faces among many objects and then judge their attractiveness, suggesting that attractive faces elicited larger P1 (100–140 ms) and P2 (150–230 ms) components compared to unattractive faces. The P1 component is a hallmark of early visual processing, reflecting the processing of low‐level features of stimuli. Such features of attractive faces may be more pronounced and therefore receive more attention. The P2 component is associated with visual encoding. Both P1 and P2 reflect the rapid and automatic perception of facial attractiveness and the analysis of low‐level structural features of faces (Schacht et al. 2008; Zhang and Deng 2012). Moreover, Marzi and Viggiano (2010) employed attractiveness rating and memory tasks, revealing that more attractive faces elicited a larger N170 (150–200 ms) compared to less attractive faces. N170 is a face‐specific component related to the structural encoding of faces. Furthermore, Wiese et al. (2014) utilized gender classification and memory tasks and discovered that attractive faces elicited a larger early posterior negativity (EPN)/N250 (270–400 ms) compared to unattractive faces. The N250 is associated with facial recognition, memory, and attention (Nasr and Esteky 2009; Neumann and Schweinberger 2009; Pierce et al. 2011; Sommer et al. 2021). The above findings indicate that attractive faces elicit stronger neural activities compared to unattractive faces, from the early visual processing stage to later social cognitive activities. Thus, attractive faces may induce larger P1, N170, P2, and N250/EPN amplitudes than unattractive faces (H1). Moreover, awareness may also influence attractiveness processing; we hypothesized that differences in brain activities between attractive and unattractive conditions could be greater in the gender judgment task compared to the CFS task (H2). On the other hand, Jiang et al. (2009) using the CFS paradigm found that fearful and neutral faces induced larger P1 and N1 amplitudes under the visible condition compared to the invisible condition. Therefore, we also examined whether the processing of general face information differed between the gender judgment task and the CFS task. We hypothesized that faces in the visible implicit task can elicit larger P1, N170, P2, and N250/EPN amplitudes compared to the invisible implicit task (H3). It is to be noted that this experiment was an exploratory study because few previous studies investigated these hypotheses.

## 2 Methods

### 2.1 Participants

The G*Power 3.1.9.7 was used to estimate the sample size according to a priori power analysis (Faul et al. 2007). At least 24 participants were required to obtain a medium effect size (*f* = 0.25) with 80% power at a significance level of 0.05. As an exploratory study, although we calculated the number of participants needed based on a medium effect size, we ultimately recruited fifty university students (23 males, 27 females) for the sake of stability of the results and potential data loss from the contrast adjustment task. Data from 3 participants was excluded from further analyses, as they could see the faces with the lowest contrast during the contrast adjustment task. Consequently, the primary analyses included data from 47 participants (22 males, *M*_age_ = 22.26 years, SD = 2.36). Additionally, based on the conventional power level (0.80) and the actual sample size (*N* = 47), we used G*Power 3.1.9.7 to do the sensitivity analysis and obtained the minimal detectable effect size of 0.17, which could be reliably detected for 66.7% of the results (see Supporting Information for review). All participants reported being physically and mentally healthy and were right‐handed, with normal or corrected‐to‐normal vision. None of them had participated in any similar experiments before. Each of them signed informed consent before the experiment. The experiment was approved by the Ethics Committee of Liaoning Normal University. All methods of this study were performed in accordance with the Declaration of Helsinki.

### 2.2 Materials

Four hundred forty‐three male faces and 296 female faces were selected from the face database of our lab (Wang et al. 2019), CUHK Face Sketch Database (CUFS; Wang and Tang 2009), and CAS‐PEAL Face Database (Gao et al. 2008). The attractiveness of the faces was determined based on ratings from 33 university students (20 females, 13 males, *M*_age_ = 21.30 years, SD = 2.14) who did not participate in the formal experiment (on a 7‐point scale from 1 = “*very unattractive*” to 7 = “*very attractive*”, 4 = “*neutral*”). Finally, 60 male faces and 60 female faces were selected as stimuli in the formal experiment. Among each sex, 30 were attractive faces and 30 were unattractive faces. A two‐way analysis of variance (ANOVA) with attractiveness and gender as factors on the attractiveness ratings revealed that the main effect of attractiveness was significant, with attractive faces (*M* = 4.31; SD = 0.62) receiving higher ratings than unattractive faces (*M* = 2.18; SD = 0.12), *F*(1, 116) = 697.47, *p* < 0.001, *η*_*p*_^*2*^ = 0.857. Neither the main effect of gender (*F*(1, 116) = 0.545, *p* = 0.462, *η*_*p*_^*2*^ = 0.005) nor the interaction between gender and attractiveness reached significance (*F*(1, 116) = 2.179, *p* = 0.143, *η*_*p*_^*2*^ = 0.018). In addition, eight faces were selected as stimuli in the contrast adjustment task and practice trials. These faces did not appear in the formal experiment.

Utilizing Photoshop 8.0.1, the 120 original face images were uniformly cropped into the same size (120 × 180 pixels) and subsequently converted to grayscale. Three groups of face images were created by reducing contrast to 30%, 20%, and 10% of the faces' original contrast. Using MATLAB R2012b, each original face image was segmented into many square grids of 8 × 8 pixels, and the grid elements were randomly rearranged as the original scrambled face image. Afterward, three contrast levels of scrambled stimuli were created by reducing contrast to 30%, 20%, and 10% of their original contrast. In addition, 20 chromatic Mondrian noises were generated using MATLAB R2012b. The size of the Mondrian images was the same as the face images. Ten of them were employed in the main CFS trials, while the other ten Mondrian images were utilized in the change detection task. In this task, the contrast of five Mondrian images was reduced by 50%.

The experimental paradigm was programmed using E‐Prime 2.0. The stimuli were presented on a 27‐in. AOC LCD monitor (1024 × 768 pixels at 100 Hz). The images on both sides of the screen were reflected to the left and right eyes of the participants using a mirror stereoscope.

### 2.3 Procedure

The experiment was carried out in a dark and quiet room. The participants were comfortably seated, and their heads were fixed on a chin rest. The formal experiment was divided into two parts, with participants doing the CFS experiment (invisible part) first and then the gender judgment experiment (visible part). EEG signals were recorded except for the contrast adjustment task. The participant's dominant eye was determined using the Dolman method (Anderson et al. 2012). The viewing distance was maintained at 1 m. We properly calibrated and adjusted the mirror stereoscope to make the pictures of the left and right visual fields on the screen fuse well in the center.

**FIGURE 1:**
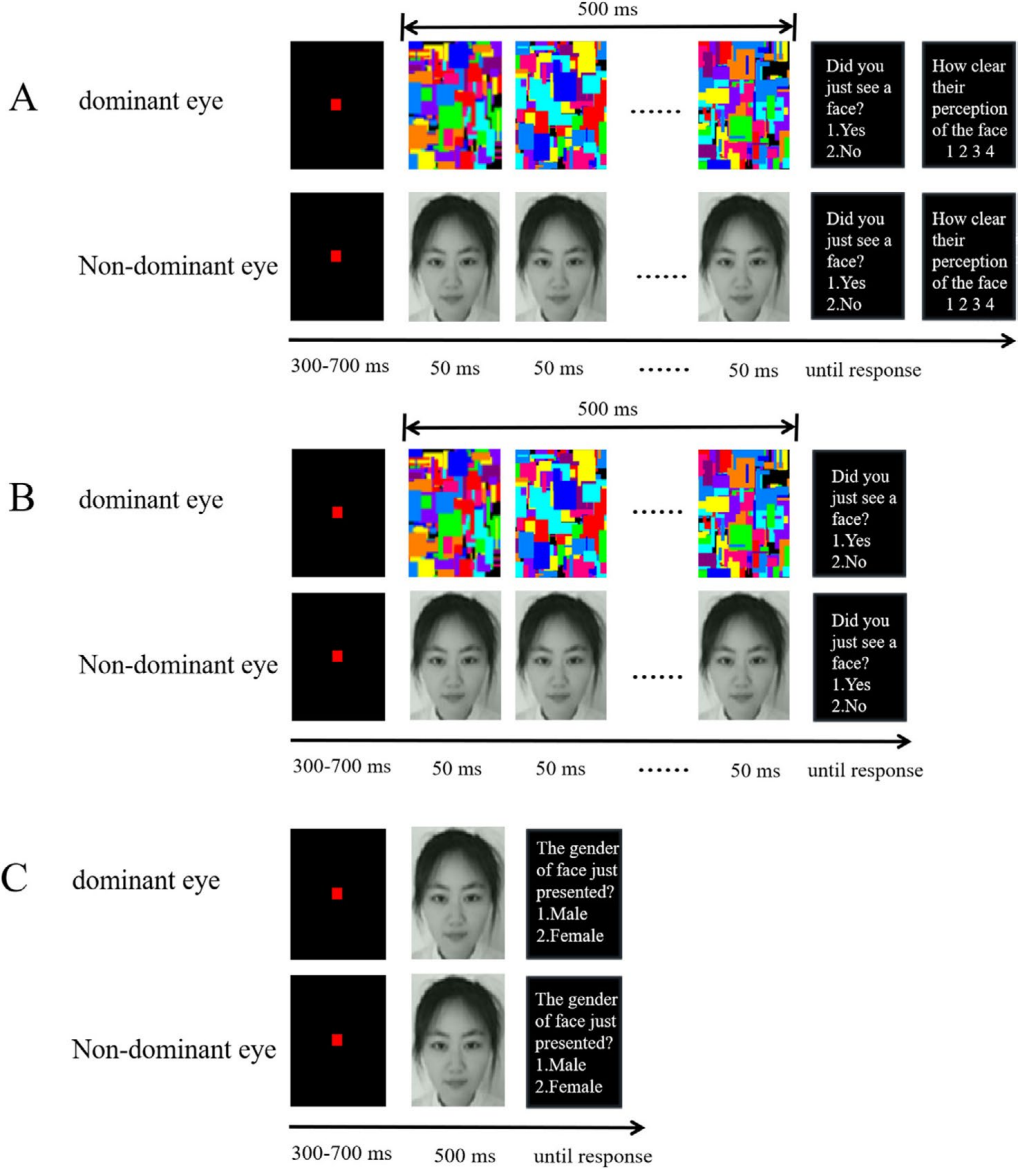
Sample stimuli and experimental procedures. (A) In the contrast adjustment task, dynamic Mondrian patterns were presented to the dominant eye, while intact faces and scrambled faces were presented to the non‐dominant eye. Participants reported whether they saw a face and how clearly they saw the face using the 4‐point PAS scale. Their performance was used to determine the proper contrast level. (B) In the continuous flash suppression (CFS) task, intact and scrambled faces presented to the non‐dominant eye with the proper contrast level were rendered invisible by Mondrian images presented to the dominant eye. Participants reported whether they saw a face and whether the brightness of the Mondrian image had darkened. (C) In the gender judgment task, intact faces were presented to both eyes of participants. Participants reported the gender of the face.

The CFS experiment was divided into two distinct phases: a contrast adjustment task and a CFS task. The contrast of the images was adjusted for each participant to ensure that the intact and scrambled faces were fully suppressed during the CFS experiment. Thus, participants were required to complete the contrast adjustment task in order to determine the proper contrast level on which the target stimuli could be rendered invisible (see Figure 1A). The contrast adjustment task included 20 trials (half comprising intact faces and the other half comprising scrambled faces). Each image was presented twice. There were 6 practice trials before the formal task. The target stimuli used in the contrast adjustment task did not appear in the CFS task. In each trial, on both the left and right visual fields, a fixation point (0.04° × 0.05°) was presented to each eye for 300–700 ms. The distance between each fixation point and the center of the screen was 9.2 cm. Then, a series of dynamic Mondrian images appeared in the dominant eye, changing every 50 ms (with a frequency of 20 Hz) for interocular suppression. Concurrent with the onset of the Mondrian images, a face image or a scrambled face image was presented to the non‐dominant eye for 500 ms. The viewing angle for both face stimuli and Mondrian images was maintained at 4.1° × 4.5°.

The contrast adjustment task started with the highest contrast level (30%). Upon the offset of the stimuli, participants were asked to answer whether they saw a face and to rate how clearly they saw the face using the 4‐point PAS scale (Ramsøy and Overgaard 2004; 1 = no experience, 2 = seeing it vaguely, 3 = seeing it almost clearly, 4 = seeing it absolutely clearly). If participants correctly reported seeing a face in 14 or more trials or reported “no experience” of the face in 14 or fewer trials, the contrast level was reduced to 20%. This procedure would repeat until participants correctly reported seeing a face in 13 or fewer trials and reported “no experience” in 15 or more trials. This contrast adjustment task was exactly the same as with Anderson et al. (2012)'s settings.

The procedure of the CFS task was the same as that of the contrast adjustment task, except that participants were not asked to complete the PAS scale at the end of each trial (see Figure 1B), and EEG signals were recorded. Before the formal CFS task, participants completed 10 practice trials. The experiment included 480 trials of intact faces, that is, each face was presented four times. Additionally, there were 120 trials of scrambled faces serving as distractors.

As suggested by Jiang et al. (2009), in the CFS paradigm, it is necessary to use a moderately demanding task to maintain participants' attention on the center of the display. Therefore, to motivate participants to concentrate on these dynamic patterns, 120 change detection trials were incorporated as a part of the CFS experiment, as inspired by Jiang et al. (2018). The procedure was mostly the same as the main CFS trials, with the following exceptions: five noise images with 50% reduced contrast and five noise images with full contrast were presented to the dominant eye. Meanwhile, only a peripheral square was presented to the non‐dominant eye. Throughout the CFS experiment, participants were instructed to judge whether the brightness of the noise image had darkened by pressing “↓” on the keyboard immediately. However, the change detection trials were discarded from further EEG analysis. In summary, the CFS experiment included 720 trials in a randomized sequence. Participants had a rest for one minute after completing every 60 trials. At the end of the CFS experiment, we replenished the brain conductive gel for each participant.

In gender judgment task (see Figure 1C), the contrast of the faces was consistent with the CFS task. In the beginning, a fixation point was presented to both eyes for 300–700 ms. Subsequently, both eyes were presented with the same face images simultaneously for 500 ms. Afterward, participants were asked to judge the gender of the face. This task consisted of 360 trials presented in a random order with 120 faces. Each face was presented three times. The formal task commenced after 6 practice trials. Participants took a rest for one minute after completing every 60 trials.

### 2.4 ERP Recording, Preprocessing, and Data Analysis

The EEG data were recorded at a sampling rate of 500 Hz with a 64‐channel system (Brain Product, Gilching, Germany) according to the 10–20 international system. The impedance was kept below 10 kΩ. Offline preprocessing was conducted using EEGLAB 2021 Tool‐box (Delorme and Makeig 2004) within MATLAB R2023b (www.mathworks.com). In the gender judgment task, data with incorrect gender judgment were excluded (4.46% of the data). In the CFS experiment, trials in which participants reported seeing faces were eliminated (1.16% of the trials). The EEG data were re‐referenced to the average of the two mastoids (TP9 and TP10) and the FCz electrode was interpolated. The data were filtered with a 0.1–30 Hz band‐pass. The EEG epochs were segmented from −100 to 500 ms around target stimuli (intact faces and scrambled faces) onset, with baseline correction using the 100 ms window before stimulus presentation. An independent component analysis (ICA) was used to remove artifacts caused by eye movements and other motions. Epochs with signals exceeding ±100 μV were excluded from the analysis. Next, epochs were averaged per electrode for each participant in each experimental condition (visible‐attractive; invisible‐attractive; visible‐unattractive; invisible‐unattractive).

**FIGURE 2:**
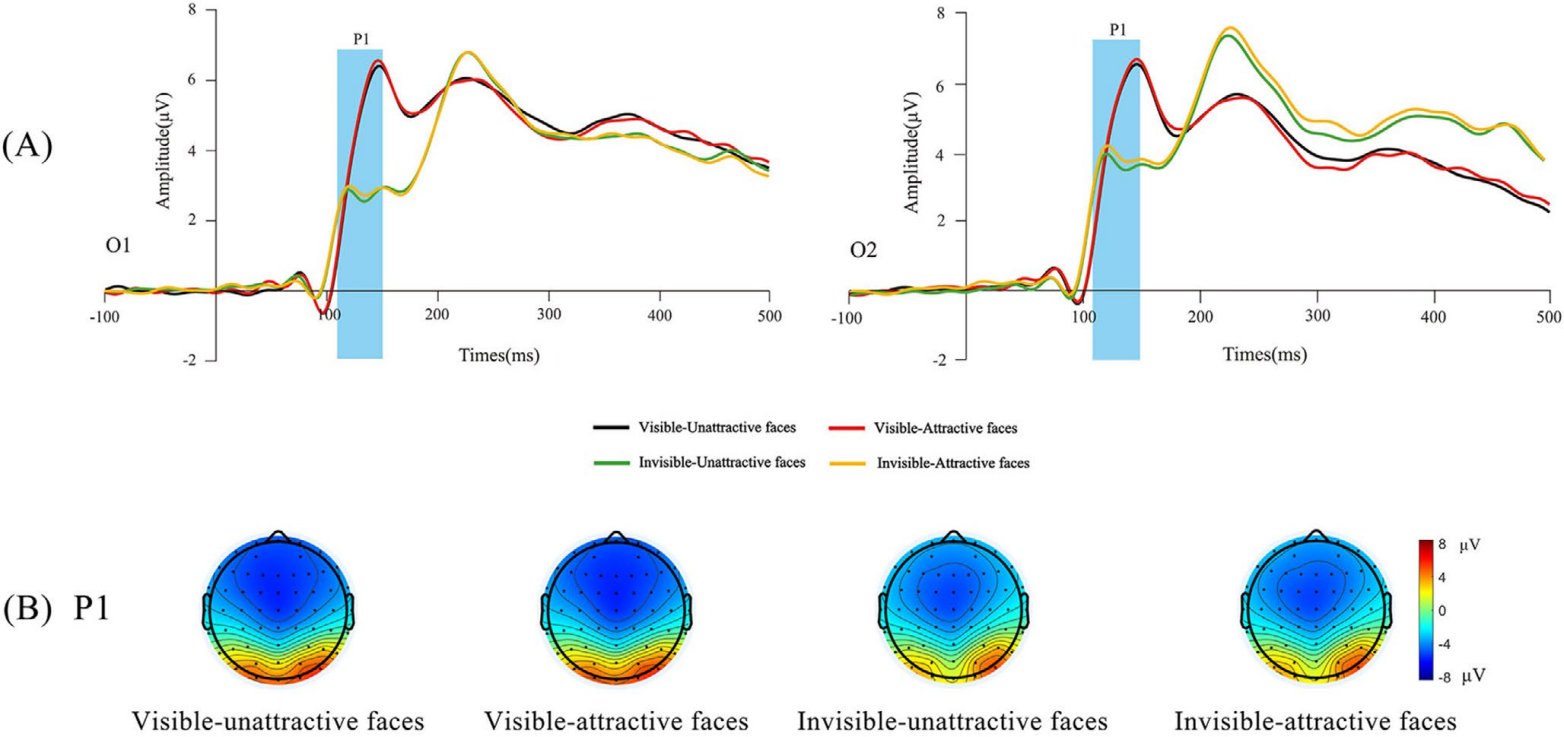
The event‐related potential (ERP) grand‐average waveforms and topographic maps of P1. (A) Grand‐average ERPs evoked by attractive and unattractive faces in different tasks (visible and invisible) at occipital electrodes. The blue highlighted areas indicate the time window for early P1 (110–150 ms). (B) The bar for four conditions of P1 topographic map ranges from −8 μV to 8 μV.

**FIGURE 3:**
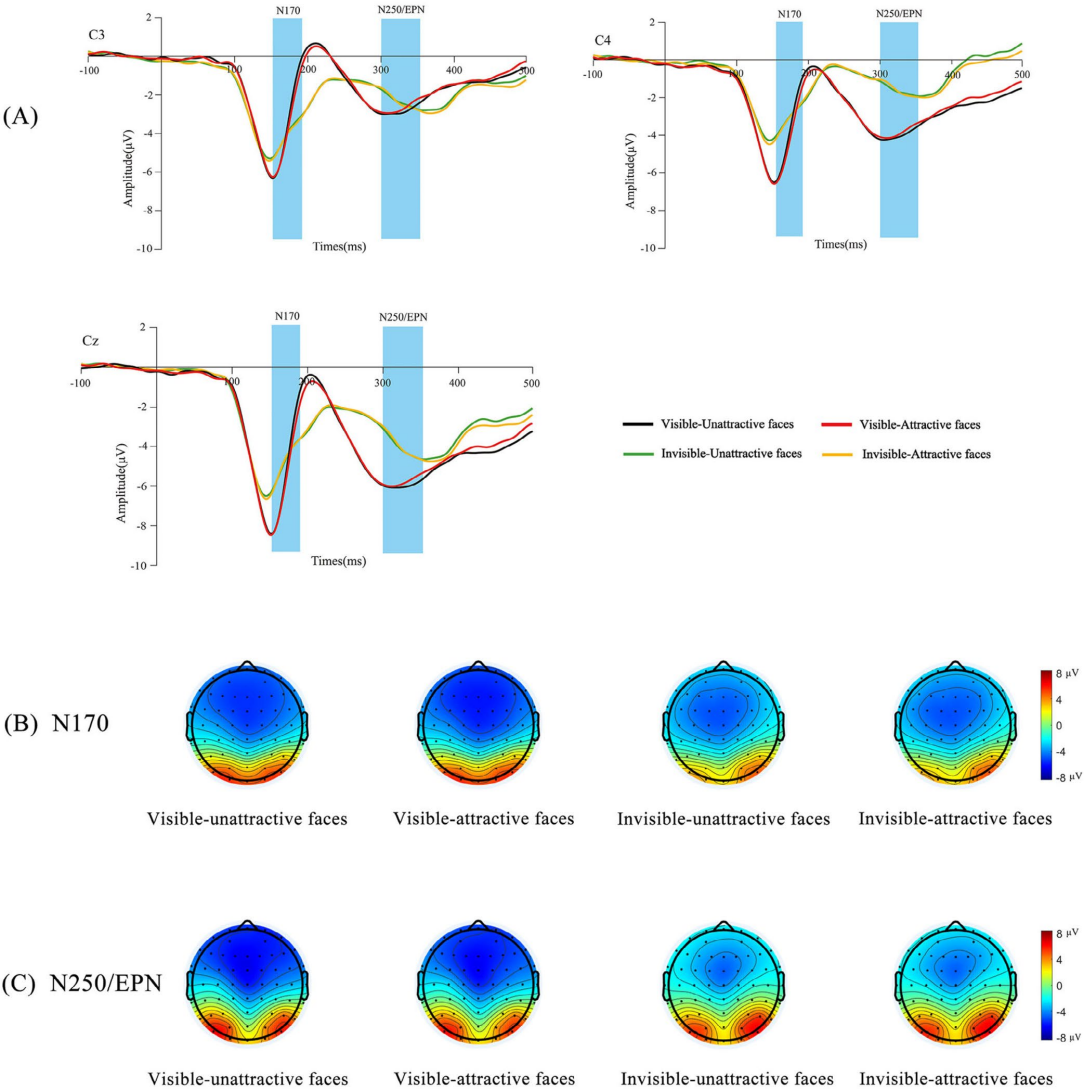
The event‐related potential (ERP) grand‐average waveforms and topographic maps of N170 and N250/Early posterior negativity (EPN). (A) Grand‐average ERPs evoked by attractive and unattractive faces in different tasks (visible and invisible) at central electrodes. The blue highlighted areas indicate time windows for N170 (150–190 ms) and N250/EPN (300–350 ms). (B) The bar for four conditions of N170 topographic map ranges from −8 μV to 8 μV. (C) The bar for four conditions of N250/EPN topographic map ranges from −8 μV to 8 μV.

**FIGURE 4:**
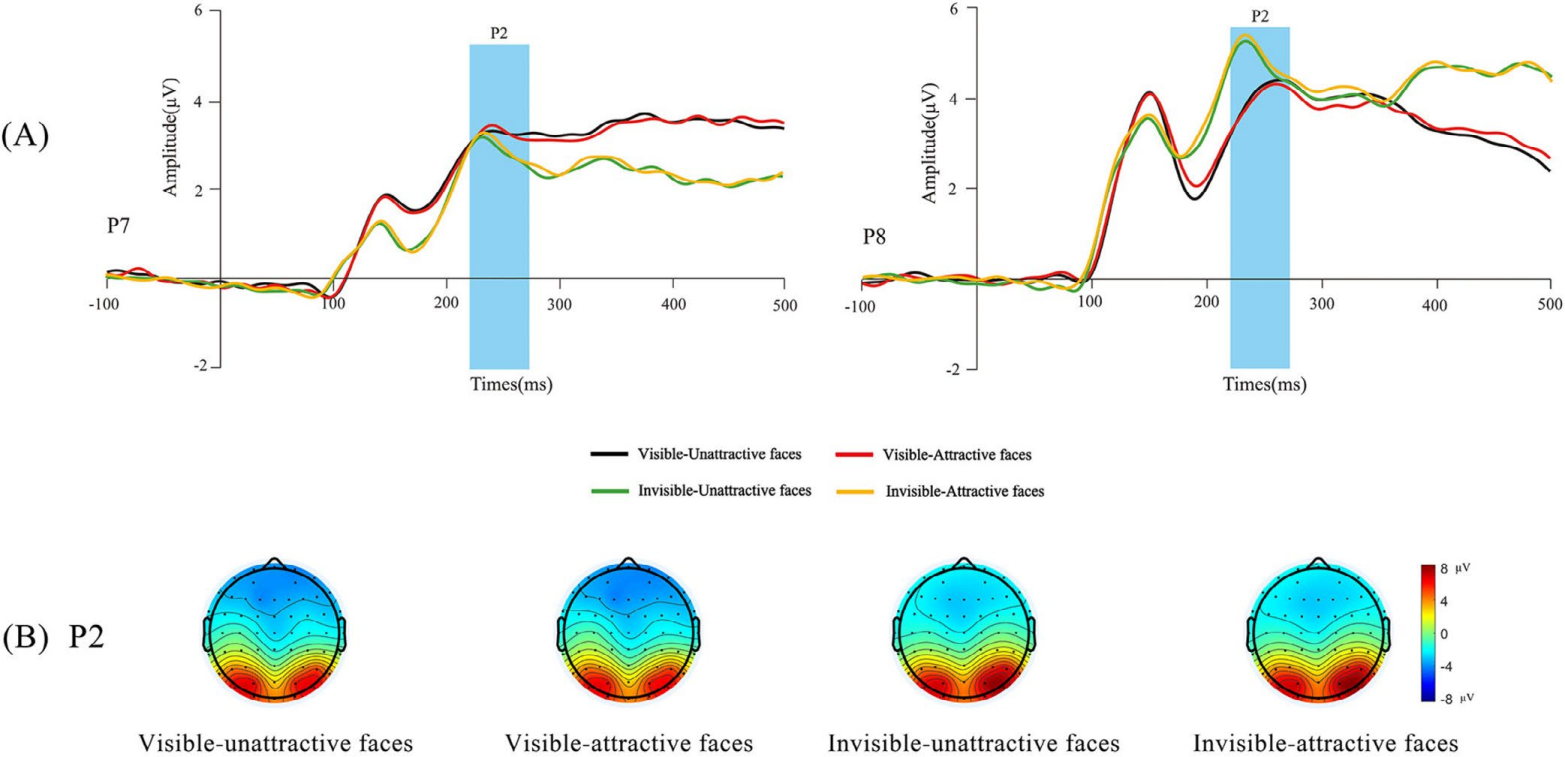
The event‐related potential (ERP) grand‐average waveforms and topographic maps of P2. (A) Grand‐average ERPs evoked by attractive and unattractive faces in different tasks (visible and invisible) at parietal electrodes. The blue highlighted areas indicate time window for P2 (220–270 ms). (B) The bar for four conditions of P2 topographic map ranges from −8 μV to 8 μV.

Based on previous studies (Wiese et al. 2014) and the waveform maps obtained in this study, the final ERP components and time windows analyzed were P1 (110–150 ms), N170 (150–190 ms), P2 (220–270 ms), and N250/EPN (300–350 ms). We selected the following electrode sites: P1 (O1, O2), N170 (C3, C4, Cz), P2 (P7, P8) and N250/EPN (C3, C4, Cz). Topographic maps of each condition are depicted in Figures 2B, 3B,C, and 4B, to further confirm the location of electrodes relative to the ERP components.

Data were analyzed using IBM SPSS Statistics 27. For each ERP component, a 2 (facial attractiveness: attractive, unattractive) × 2 (visibility: visible, invisible) repeated measures ANOVA was performed on the mean wave amplitudes. Greenhouse–Geisser correction was used when the assumption of sphericity was not met.

## 3 Results

### 3.1 P1

As can be seen from Figure 2A, the main effect of facial attractiveness was significant, *F*(1, 46) = 4.256, *p* = 0.045, *η*_*p*_^2^ = 0.085, such that attractive faces (*M* = 4.067 μV, SD = 0.370 μV) induced a larger P1 than unattractive faces (*M* = 3.939 μV, SD = 0.373 μV). The main effect of visibility was also significant, *F*(1, 46) = 20.484, *p* < 0.001, *η*_*p*_^2^ = 0.308. The visible condition (*M* = 4.864 μV, SD = 0.394 μV) induced a larger P1 than the invisible condition (*M* = 3.142 μV, SD = 0.438 μV). The interaction between facial attractiveness and visibility was not significant, *F*(1, 46) = 0.004, *p* = 0.951, *η*_*p*_^2^ < 0.001.

### 3.2 N170

As can be seen from Figure 3A, the main effect of facial attractiveness was significant, *F*(1, 46) = 5.615, *p* = 0.022, *η*_*p*_^2^ = 0.109, such that attractive faces (*M* = −4.488 μV, SD = 0.385 μV) induced a larger N170 than unattractive faces (*M* = −4.287 μV, SD = 0.399 μV). The main effect of visibility was not significant, *F*(1, 46) = 3.99, *p* = 0.052, *η*_*p*_^2^ = 0.08. The interaction between facial attractiveness and visibility was significant, *F*(1, 46) = 4.071, *p* = 0.049, *η*_*p*_^2^ = 0.081. Simple effect analyses indicated that under the visible condition, attractive faces (*M* = −4.836 μV, SD = 0.415 μV) elicited a larger N170 than unattractive faces (*M* = −4.466 μV, SD = 0.453 μV), *F*(1, 46) = 7.489, *p* = 0.009, *η*_*p*_^2^ = 0.14. For attractive faces, the visible condition (*M* = −4.836 μV, SD = 0.415 μV) elicited a larger N170 than the invisible condition (*M* = −4.139 μV, SD = 0.398 μV), *F*(1, 46) = 7.093, *p* = 0.011, *η*_*p*_^2^ = 0.134. Other simple effects were not significant, *F*s ≤ 1.506, *p*s ≥ 0.226.

### 3.3 P2

As can be seen from Figure 4A, the main effect of facial attractiveness was not significant, *F*(1, 46) = 0.402, *p* = 0.529, *η*_*p*_^2^ = 0.009. The main effect of visibility was also not significant, *F*(1, 46) = 1.847, *p* = 0.181, *η*_*p*_^2^ = 0.039. The interaction between facial attractiveness and visibility was not significant, *F*(1, 46) = 1.559, *p* = 0.218, *η*_*p*_^2^ = 0.033.

### 3.4 N250/EPN

As can be seen from Figure 3A, the main effect of facial attractiveness was not significant, *F*(1, 46) = 0.691, *p* = 0.410, *η*_*p*_^2^ = 0.015. The main effect of visibility was significant, *F*(1, 46) = 12.246, *p* < 0.001, *η*_*p*_^2^ = 0.210. The visible condition (*M* = −4.229 μV, SD = 0.564 μV) induced a larger N250/EPN than the invisible condition (*M* = −2.650 μV, SD = 0.469 μV). The interaction between facial attractiveness and visibility was not significant, *F*(1, 46) = 0.500, *p* = 0.483, *η*_*p*_^2^ = 0.011.

## 4 Discussion

The present study compared differences in neural activities evoked by facial attractiveness with different input intensities by presenting faces in two implicit tasks: a CFS task (invisible condition) and a gender judgment task (visible condition).

### 4.1 Processing of Facial Attractiveness

Firstly, attractive faces elicited larger P1 amplitudes compared to unattractive faces regardless of the visibility of faces. The P1 component reflects the processing of facial features and structure (Zhang et al. 2016), indicating that attractive faces were differentiated from unattractive faces between 110 ms and 150 ms. This finding suggests that invisible face images are processed to the level where the brain can tell an attractive face from an unattractive face, further indicating the automaticity of attractiveness appraisal and the reward value of attractive faces (Aharon et al. 2001). As facial attractiveness is important in evolution, people could still perceive attractiveness information in the early stage even without conscious representation. This finding is also in line with b‐CFS studies (Hung et al. 2016; Nakamura and Kawabata 2018); when attractive and unattractive faces were paired with identical suppression noise, attractive faces took less time to gain dominance compared to unattractive faces. Moreover, attractive faces elicited larger P1 amplitudes in the gender judgment task. This is consistent with some research in which facial attractiveness was task‐irrelevant, e.g., economic decision‐making toward the faces (Ma et al. 2015, 2017; Pei and Meng 2018). However, other studies using the gender judgment task did not reveal an effect of facial attractiveness on P1 (Schacht et al. 2008; Wiese et al. 2014). The discrepancies might arise from the control of stimuli since Wiese et al. controlled the uniqueness between attractive and unattractive faces, albeit we did not. Schacht et al. (2008) did not control the emotion of faces which may confound with the attractiveness effect.

Secondly, attractive faces elicited larger N170 amplitudes compared to unattractive faces only when the faces were visible. This is consistent with previous research using an old/new recognition task (Zhang and Deng 2012). N170 is associated with facial structure coding and face classification processing (Marzi and Viggiano 2010). Processing of the reward value of faces is also associated with N170 (Rellecke et al. 2012). Previous studies have also found that aesthetic processing of faces modulates the N170 component (Pizzagalli et al. 2002), and attractive faces evoke larger N170 in an attractiveness judgment task (Marzi and Viggiano 2010). Similar to prior research, our finding reflects the fact that attractiveness information can be distinguished early and implicitly in facial processing. However, when faces were rendered invisible by CFS, N170 was not different between attractive and unattractive conditions, suggesting processing of facial attractiveness differs depending on visibility. As CFS is a robust method to keep stimuli invisible, neither P2 nor N250/EPN amplitude was influenced by facial attractiveness, suggesting that CFS eliminated the processing of facial attractiveness. This is similar to prior research using a masking paradigm to render faces invisible (Hou et al. 2023).

It is noteworthy that the processing of facial attractiveness was weaker in the current study compared with previous research. The effect of facial attractiveness was absent on P2 and N250/EPN in the gender judgment task. However, previous studies have consistently reported that the influence of facial attractiveness extends to later ERP components under the visible condition (Marzi and Viggiano 2010; Wiese et al. 2014; Zhang et al. 2011). The differences may stem from the following reasons: Firstly, the difference in ratings between attractive and unattractive faces may not be large enough (2.13) so that the facial attractiveness effect is weak. Secondly, compared to other studies, the contrast of the faces in the present study was lower to fit the settings of the CFS paradigm. To be comparable between invisible and visible conditions, the contrast of faces in the gender judgment task was as low as that in the CFS task, which is too weak to capture attention, resulting in the absence of the facial attractiveness effect in the later components. Thirdly, the tasks used in the present study were different from most of the tasks in previous research, such as some studies that found the facial attractiveness effect on the P2 component using an attractiveness judgment and a covert orienting task (Zhang and Deng 2012; Van Hooff et al. 2011) and other studies that observed the N250/EPN differences using attractiveness rating, classification, or memory tasks (Marzi and Viggiano 2010; Werheid et al. 2007). Studies that also used the gender judgment task as this study reported facial attractiveness effects on the EPN rather than the P2 component (Schacht et al. 2008; Wiese et al. 2014). As mentioned previously, the present study's control of the stimuli differed somewhat compared to these studies, which may cause the difference.

### 4.2 General Processing of Visible and Invisible Faces

We also examined whether CFS reduced general face processing (for both attractive and unattractive faces). Despite the same faces being presented for the same duration in both the gender judgment task and the CFS task, some brain activities were greatly reduced by interocular suppression, though still measurable, such that larger P1 (110–150 ms) and N250/EPN (300–350 ms) amplitudes were evoked in the visible condition compared to the invisible condition. This demonstrates that the CFS paradigm is highly effective in rendering the stimuli invisible (Tsuchiya and Koch 2005). This is also similar to the findings of Jiang et al. (2009) that fearful and neutral faces elicited larger P1 amplitudes in the visible condition compared to the invisible condition. It is worth noting that P2 amplitude was not affected by visibility. P2 is associated with early attentional allocation, responding to early perceptual processing of face structure and features (Van Hooff et al. 2011; Marzi and Viggiano 2010; Chen et al. 2012). This finding suggests that general facial information can indeed be processed to some extent in the absence of visual awareness.

Additionally, when the faces were unattractive, N170 was not different between visible and invisible conditions. However, N170 amplitude was greater under the visible condition compared to the invisible condition when the faces were attractive. This is consistent with Jiang et al. (2009), which reported greater N170 for fearful and neutral faces under the visible condition compared to the invisible condition. It may be because the reward value of unattractive faces is not yet large enough; they fail to gain attention even in the visible condition, resulting in null influence of visual awareness on brain activities.

There were some limitations in this study. We acknowledge that prolonged EEG experiments pose many potential problems, such as drying of the conductive gel, which in turn leads to unstable EEG signals, increased impedance between the electrodes and the scalp, reduced signal‐to‐noise ratio (SNR), and many other problems that affect the collection and quality of experimental data. In addition, a long duration makes the participants too tired, which in turn affects the data. Moreover, to avoid participants guessing the purpose of the experiment, the CFS task was in front of the gender judgment task, but this may lead to a carryover effect. Future research should control the length of the experiment and employ a counterbalanced design to test the findings of this experiment.

## 5 Conclusions

This experiment manifested that attractive faces were implicitly differentiated from unattractive faces between 110 and 150 ms (P1), indicating that facial attractiveness can be automatically processed regardless of the conscious representation of faces. The effect of attractiveness continued to 150–190 ms (N170) only in the visible condition, while the effect disappeared later on. N170 amplitude was greater under the visible condition compared to the invisible condition when the faces were attractive. Furthermore, face processing under the visible condition caused larger amplitudes of P1 and N250/EPN components compared to the invisible condition. The findings indicate that although the intensity of visual input has an impact on facial processing, the brain encodes facial attractiveness in the early stage implicitly even when the faces are rendered invisible.

## Ethics Statement

The research was conducted according to the guidelines of the Declaration of Helsinki and approved by the Ethics Committee of Liaoning Normal University (Project identification code: 31400869 and approval date: 12 December 2016). Informed consent was obtained from all participants involved in the study.

## Conflicts of Interest

The authors declare no conflicts of interest.

## Supporting information


Data S1.
