# Supplementary material for: Invisible and Visible Processing of Facial Attractiveness in Implicit Tasks: Evidence From Event‐Related Potentials (ERPs)
Source: Psych J. 2025 Apr 23;14(4):573–82. doi: 10.1002/pchj.70014 (PMC12318587; doi:10.1002/pchj.70014)
Supplement: Supplementary file 1 — Data S1. [file PCHJ-14-573-s001.docx]

**Sensitivity analysis**

*Following the suggestion from Reviewers, we used G*Power 3.1.9.7 to do the sensitivity analysis, based on the conventional power level (0.80) and the actual sample size (N=47). We obtained the minimal detectable effect size (f-value) of 0.17. The minimal value of η_p_^2^ was calculated from the conversion formula for η_p_^2^ and f-values: f =* $\sqrt{\frac{\eta p2}{1-\eta p2}}$ *, η_p_^2^ = 0.028.*

*We then used IBM SPSS Statistics 27 to analyze the ERP data (N=47) and calculate η_p_*^2^ *of the main effects and interactions on each ERP component. The f-values (see* ***Table 1****) were also calculated from the conversion formula for η_p_*^2^ *and f-values: f =* $\sqrt{\frac{\eta p2}{1-\eta p2}}$ *.*

***Table 1 η_p_*^2^ *and f-values (including main effects and interactions) based on the 47 total data sets***

| *ERP Components* | *Facial attractiveness-η_p_*^2^ | *Facial attractiveness-f* | *Visibility-η_p_*^2^ | *Visibility-f* | *Facial attractiveness×Visibility-η_p_*^2^ | *Facial attractiveness×Visibility-f* |
| --- | --- | --- | --- | --- | --- | --- |
| *P1* | *0.085* | *0.30* | *0.308* | *0.67* | *< 0.001* | *0.00* |
| *N170* | *0.109* | *0.35* | *0.080* | *0.29* | *0.081* | *0.30* |
| *P2* | *0.009* | *0.10* | *0.039* | *0.20* | *0.033* | *0.18* |
| *N250/EPN* | *0.015* | *0.12* | *0.210* | *0.52* | *0.011* | *0.11* |

*In comparison between our results and minimal detectable effect size, it can be seen that 41.7% of the results reached the minimum effect size (η_p_^2^ = 0.028) and were significant. 25% of the results reached the minimum effect size (η_p_^2^ = 0.028) but were not significant. The remaining 33.3% of the results did not reach the minimum effect size (η_p_^2^ = 0.028) and were not significant. This helps to interpret non-significant findings. With this sample, an effect size of 0.17 could be reliably detected for 66.7% of the effects, suggesting the amount of participants used in this study is reasonable.*
